# Germinated Rice Seeds Improved Resveratrol Production to Suppress Adipogenic and Inflammatory Molecules in 3T3-L1 Adipocytes

**DOI:** 10.3390/molecules28155750

**Published:** 2023-07-29

**Authors:** Chaiwat Monmai, Jin-Suk Kim, So-Hyeon Baek

**Affiliations:** Department of Agricultural Life Science, Sunchon National University, Suncheon 59722, Republic of Korea; bbuayy@gmail.com (C.M.); kimjs6911@naver.com (J.-S.K.)

**Keywords:** resveratrol, germination, PPARγ, C/EBPα, ERK, Akt, oil red o, cytokine, 3T3-L1 cells

## Abstract

Obesity is a major risk factor for a variety of diseases and contributes to chronic inflammation. Resveratrol is a naturally occurring antioxidant that can reduce adipogenesis. In this study, the antiadipogenic and anti-inflammatory activities of resveratrol-enriched rice were investigated in 3T3-L1 adipocyte cells. Cotreatment of dexamethasone and isobutylmethylxanthin upregulated adipogenic transcription factors and signaling pathways. Subsequent treatment of adipocytes with rice seed extracts suppressed the differentiation of 3T3-L1 by downregulating adipogenic transcription factors (peroxisome proliferator-activated receptor γ and CCAAT/enhancer-binding protein α) and signaling pathways (extracellular signal-regulated kinase 1/2 and protein kinase B Akt), this was especially observed in cells treated with germinated resveratrol-enriched rice seed extract (DJ526_5). DJ526_5 treatment also markedly reduced lipid accumulation in the cells and expression of adipogenic genes. Lipopolysaccharide (LPS)-induced inflammatory cytokines (prostaglandin-endoperoxide synthase 2 (*COX-2*), tumor necrosis factor (*TNF*)*-α*, interleukin (*IL*)*-1β*, and *IL-6*) decreased in cells treated with DJ526_5. Collectively, DJ526_5 exerts antiadipogenic effects by suppressing the expression of adipogenesis transcription factors. Moreover, DJ526_5 ameliorates anti-inflammatory effects in 3T3-L1 adipocytes by inhibiting the activation of phosphorylation NF-κB p65 and ERK ½ (MAPK). These results highlight the potential of resveratrol-enriched rice as an alternative obesity-reducing and anti-inflammatory agent.

## 1. Introduction

Over-accumulated energy can be stored as lipids in the body and lead to obesity [1,2], a risk factor for diabetes [3], cardiovascular diseases, cancer [4,5], respiratory diseases [6], and prostate diseases [7]. Adipogenesis, the process by which preadipocytes differentiate into mature adipocytes, is linked to several signaling pathways [8], particularly the extracellular signal-regulated kinase 1/2 (ERK 1/2) and protein kinase B (Akt) signaling pathways [9,10]. The morphological transition of fibroblast-like cells into rounder adipocytes [8,11] is triggered by dexamethasone, isobutylmethylxanthin, and insulin [12,13]. Dexamethasone and isobutylmethylxanthin (IBMX) are direct inducers of adipogenesis, whereas insulin stimulates the cells to take up glucose [8,14]. The main transcription factors that regulate adipogenesis are peroxisome proliferator-activated receptor γ (PPARγ) and CCAAT/enhancer-binding protein α (C/EBPα) [15,16,17]; their activation upregulates adipogenic genes such as glucose transport member 4 (GLUT-4), fatty acid synthase (FAS), adiponectin, stearoyl-CoA desaturase, leptin, and acetyl-CoA carboxylase [18,19,20].

Recently, several studies have shown that proinflammatory cytokines such as interleukin (IL)-1β, IL-6, and tumor necrosis factor-α (TNF-α) in adipocytes and macrophages contribute to the onset and progression of obesity [21,22,23]. Obesity upregulates the NF-κB [24] and ERK [25] signaling pathways in adipocytes, thereby increasing the production of proinflammatory molecules. The production of proinflammatory cytokines by adipocyte hypertrophy and hyperplasia led to adipocyte tissue inflammation [26]. The inhibition of the NF-κB and ERK signaling pathways causes a reduction in adipocyte inflammation [27]. Therefore, the bioactive compounds that reduce adipogenic and obesity-induced inflammation are being searched for.

Resveratrol (3,5,4′-trihydroxy-trans-stilbene) is a polyphenolic compound found in certain plants and foods like peanuts, red wine, grapes, and mulberries [28,29,30,31]. Resveratrol may combat aging, inflammation, and the onset of cardiovascular diseases and cancer [32,33,34,35,36,37,38]. Resveratrol-producing rice was previously generated by introducing the stilbene synthase (*STS*) of *Arachis hypogaea* variety Palkwang into Dongjin rice, *Oryza sativa* japonica [39]. This transgenic rice accumulates resveratrol in its grains. Unlike Dongjin (DJ) rice seed extract, the resveratrol-producing rice seed (DJ526) exerted anti-inflammatory effects in LPS-stimulated RAW264.7 cells, and the germination can increase resveratrol content in rice seeds [40]. Here, the antiadipogenic and anti-inflammatory effects of germinated resveratrol-producing rice were evaluated in 3T3-L1 adipocytes.

## 2. Results

### 2.1. Effect of Germinated Rice Seed Extracts on Cell Viability

**Figure 1:**
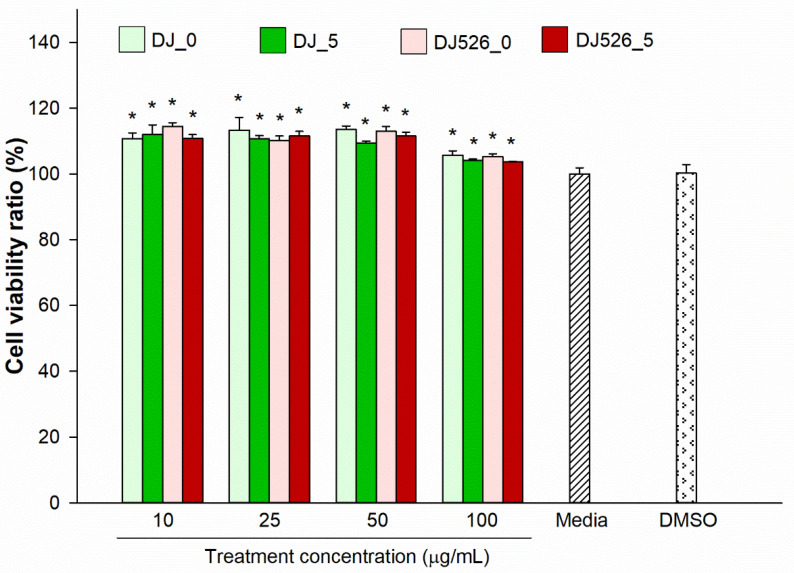
Effect of germinated rice seed extracts on cell viability, which was determined relative to the untreated group (media). The concentration of DMSO was 0.1%. Significant differences at *p* < 0.05 (*) were determined by comparing treatment outcomes with the DMSO group.

The cytotoxicity of the treatments was evaluated using an EZ-Cytox cell viability kit. Cell viability was evaluated in response to various concentrations of each treatment and compared to the untreated group (media). Treatments ranging from 10 to 100 µg/mL DJ_0, DJ_5, DJ526_0, and DJ526_5 significantly enhanced cell proliferation compared to the DMSO group (Figure 1). There was no significant difference in cell viability between 0.1% DMSO-treated cells and the control group (Media). No cytotoxic effects were observed in 3T3-L1 cells for concentrations as high as 100 µg/mL.

### 2.2. Effect of Germinated Rice Seed Extracts on Cell Differentiation

**Figure 2:**
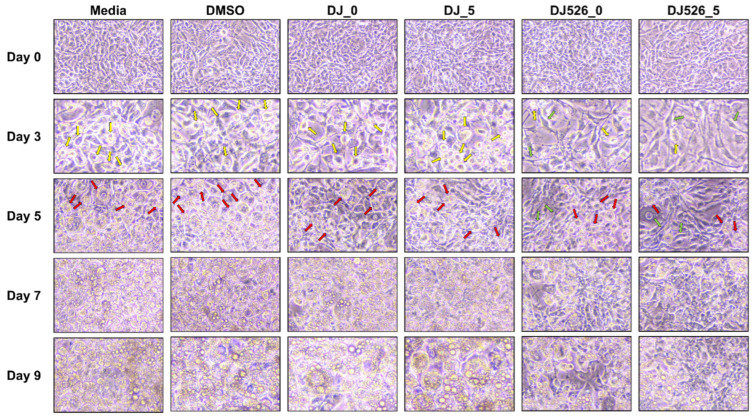
Representative microscopy images of 3T3-L1 cell differentiation from Day 0 to Day 9 post-induction. The concentrations of treatments and DMSO are 100 µg/mL and 0.1%, respectively. Green arrows represent the fibroblast-like cell shape during the differentiation process. Yellow arrows represent the round shape of cells. Red arrows represent the lipid-containing cells.

The effect of rice seed extracts on cell differentiation was monitored from 0 days post treatment (dpt) to9 dpt. Before induction (0 dpt), most cells resembled fibroblasts (Figure 2). At 3 dpt, the cells became visibly rounder, and the number of rounded cells increased at 5 dpt. Lipid droplets were observed in the cells, and more than half of the media- and DMSO-treated cells exhibited lipid droplets at 5 dpt. Some lipid droplets were observed in DJ526_0- and DJ526_5-treated cells at 5 dpt. At 7 dpt, lipid droplets were observed in most media- and DMSO-treated cells and further increased at 9 dpt. However, DJ526_0 and DJ526_5 treatments reduced the number of lipid-containing cells. At 7 dpt, lipid droplets were found in less than 50% of DJ526_0- and DJ526_5-treated cells. Unlike cells treated with media or DMSO, DJ526_5-treated cells showed delayed and reduced accumulation of lipid droplets.

### 2.3. Effect of Germinated Rice Seed Extracts on Lipid Accumulation in Cells

**Figure 3:**
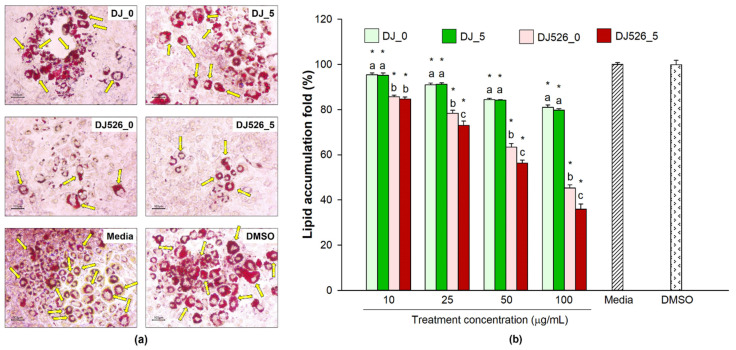
Effect of germinated rice seed extracts on lipid accumulation in adipocytes. (**a**) Representative microscopy image of cells stained with oil red O (scale bar = 100 µm; treatment concentration is 100 µg/mL) and (**b**) lipid accumulation in treated adipocytes compared to the untreated group (media). The concentration of DMSO was 0.1%. Significant differences at *p* < 0.05 (*) were determined in comparison to the DMSO group. A one-way ANOVA followed by post hoc Duncan’s multiple range tests was used to determine the difference between treatments. Letters (a–c) indicate significant differences (*p* < 0.05) between the DJ_0, DJ_5, DJ526_0, and DJ526_5 groups at the same concentration (where a > b > c). In the statistical analysis, “a” represents the reference group, “b” represents significantly lower than the “a” group (*p* < 0.05), and “c” represents significantly lower than the “b” group (*p* < 0.05). Yellow arrows represent the lipids located inside adipocytes.

At 9 dpt, the cells were stained with oil red O solution to assess lipid accumulation, whose fold change was calculated by comparing the lipid accumulation of the treatment group to that of the untreated group (media). As shown in Figure 3, treatment with 0.1% DMSO and media elicited similar levels of lipid accumulation in adipocytes (red spots). However, treatment with rice seed extracts (DJ_0, DJ_5, DJ526_0, or DJ526_5) significantly decreased lipid accumulation in the cells (*p* < 0.05). Lipid accumulation decreased with increasing treatment concentration. The greatest inhibition of lipid accumulation was observed in the DJ526_5-treated cells (*p* < 0.05).

### 2.4. Effect of Germinated Rice Seed Extracts on Adipogenic Gene Expression

**Figure 4:**
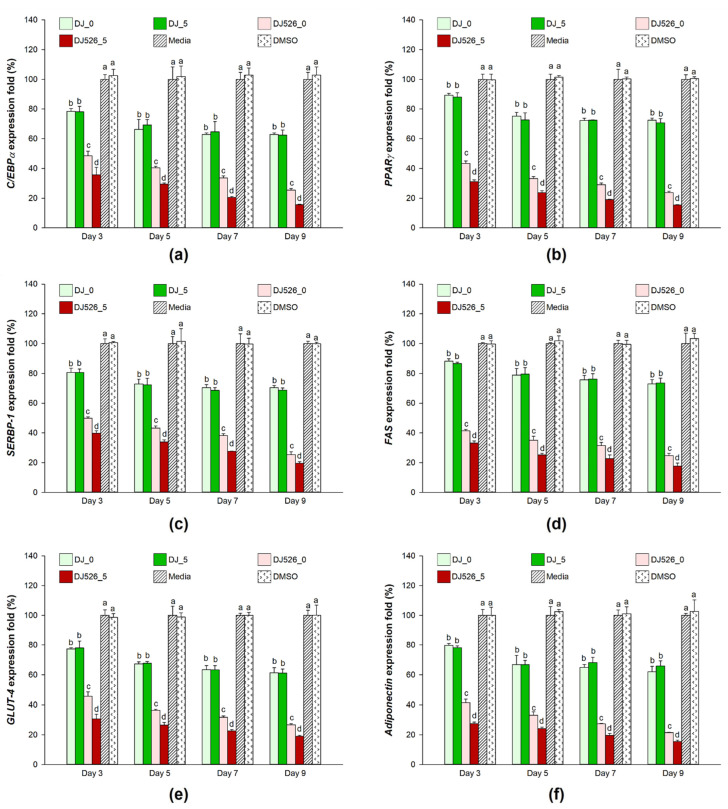
Effect of treatment with germinated rice seed extracts on expression of adipogenic genes: (**a**) *C/EBPα*, (**b***) PPARγ*, (**c**) *SERBP-1*, (**d**) *FAS*, (**e**) *GLUT-4*, and (**f**) *adiponectin*. The concentrations of treatments and DMSO were 100 µg/mL and 0.1%, respectively. A one-way ANOVA followed by post hoc Duncan’s multiple range tests was used to determine the difference between treatments. Letters (a–d) indicate significant differences (*p* < 0.05) between DJ_0, DJ_5, DJ526_0, and DJ526_5 on the same treatment day (where a > b > c > d). In the statistical analysis, “a” represents the reference group, “b” represents significantly lower than the “a” group (*p* < 0.05), “c” represents significantly lower than the “b” group (*p* < 0.05), and “d” represents significantly lower than the “c” group (*p* < 0.05).

The expression levels of adipogenic transcription factors and genes were measured in rice seed extract-treated cells during differentiation. MDI media induced the expression of adipogenic transcription factors (*C/EBPα*, *PPARγ*, and *SERBP-1*) and genes (*FAS*, *GLUT-4*, and adiponectin) (Figure 4). The expression levels of these genes were upregulated from 3 dpt to 9 dpt. However, treatment with rice seed extracts significantly reduced expression levels of these genes at 3 dpt (early stage of adipogenesis) and continued to suppress them until 9 dpt. Unlike normal rice seed extracts (DJ_0 and DJ_5), DJ526_0 and DJ526_5 rice seed extracts markedly downregulated adipogenic factors as well as fat-forming genes; their mRNA expression levels were also lower in DJ526_5-treated cells in comparison to DJ526_0-treated cells. Overall, treatment with resveratrol-producing rice seed extracts significantly decreased the expression of adipogenesis-related genes. Moreover, germination strongly suppressed the expression of adipogenic genes by increasing resveratrol content.

### 2.5. Effect of Germinated Rice Seed Extracts on Expression of Adipogenic Proteins

**Figure 5:**
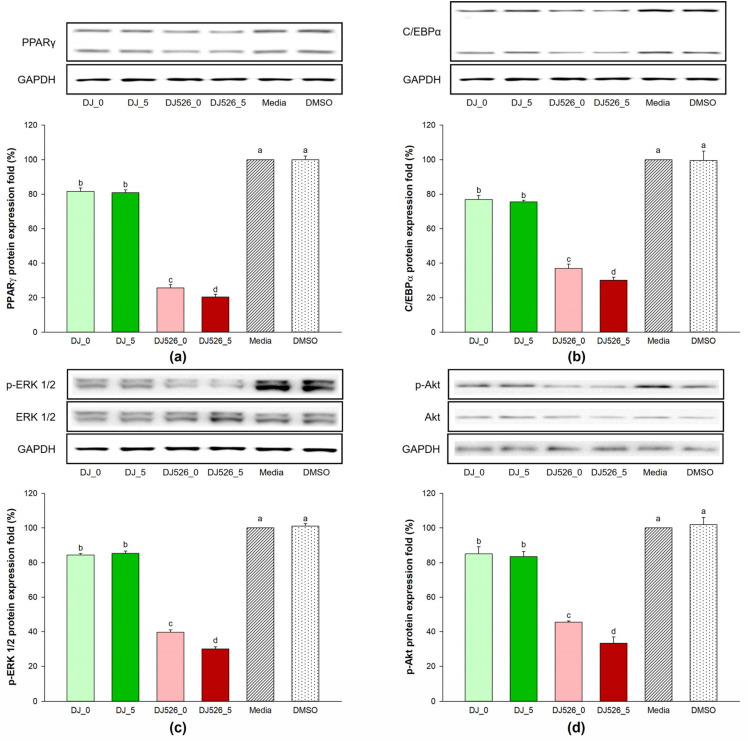
Effect of treatment with germinated rice seed extracts on expression of proteins related to adipogenesis: (**a**) PPARγ, (**b**) C/EBPα, (**c**) p-ERK 1/2, and (**d**) p-Akt. The concentrations of treatments and DMSO were 100 µg/mL and 0.1%, respectively. A one-way ANOVA followed by post hoc Duncan’s multiple range tests was used to determine the difference between treatments. Letters (a–d) indicate significant differences (*p* < 0.05) between DJ_0, DJ_5, DJ526_0, and DJ526_5 (where a > b > c > d). In the statistical analysis, “a” represents the reference group, “b” represents significantly lower than the “a” group (*p* < 0.05), “c” represents significantly lower than the “b” group (*p* < 0.05), and “c” represents significantly lower than the “d” group (*p* < 0.05).

As shown in Figure 5, MDI media promoted the production of adipogenic transcription factor proteins in the untreated and DMSO groups. The expression levels of adipogenic transcription factors, PPARγ and C/EBPα, markedly decreased in the DJ_0-, DJ_5-, DJ526_0-, and DJ526_5-treated cells. Additionally, treatment with DJ_0, DJ_5, DJ526_0, and DJ526_5 decreased the expression levels of proteins (phosphorylated (p)-ERK 1/2 and p-Akt) linked to adipogenesis. The expression levels of PPARγ, C/EBPα, p-ERK 1/2, and p-Akt were also downregulated in cells treated with high concentrations of resveratrol (DJ526_5) in comparison with those treated with normal rice (DJ_0 and DJ_5) or nongeminated resveratrol-producing rice (DJ526_0).

### 2.6. Effect of Germinated Rice Seed Extracts on Viability of LPS-Stimulated Adipocytes

**Figure 6:**
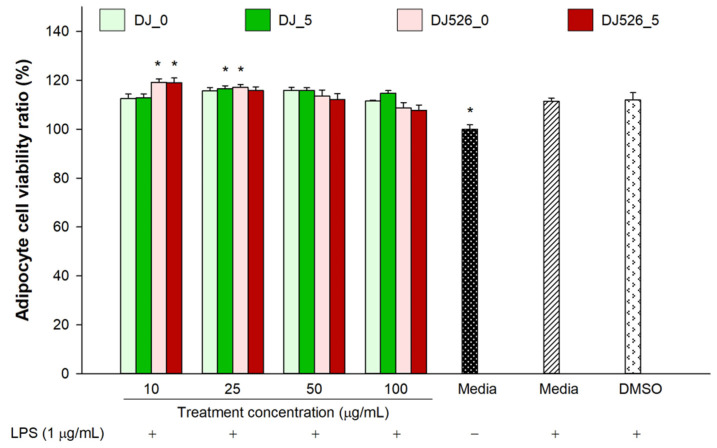
Effect of germinated rice seed extracts on viability of LPS-stimulated adipocytes. The cell viability ratio was determined relative to the untreated group (media (−)). The concentration of DMSO was 0.1%. For LPS treatment, “+” represents the treatment of LPS and “−” represents the nontreatment of LPS. Significant differences (*) were determined via comparison with the DMSO group (*p* < 0.05).

Compared to unstimulated cells (media (−)), adipocytes cultured in media (+) and DMSO proliferated significantly in response to 1 µg/mL of LPS (Figure 6). Compared to the DMSO group, cells treated with DJ_0, DJ_5, DJ526_0, and DJ526_5 exhibited similar or higher levels of viability. No cytotoxic effects were observed in adipocytes treated with DJ_0, DJ_5, DJ526_0, and DJ526_5 at concentrations from 10 to 100 µg/mL.

### 2.7. Effect of Germinated Rice Seed Extracts on Expression of Inflammatory Genes

**Figure 7:**
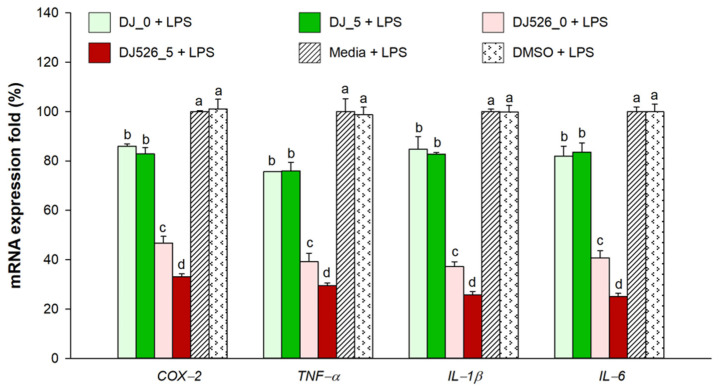
Effect of germinated rice seed extracts on proinflammatory gene expression in LPS-stimulated adipocytes. The concentrations of treatments and DMSO were 100 µg/mL and 0.1%, respectively. A one-way ANOVA followed by post hoc Duncan’s multiple range tests was used to determine the difference between treatments. Letters (a–d) indicate significant differences (*p* < 0.05) between DJ_0, DJ_5, DJ526_0, and DJ526_5 (where a > b > c > d). In the statistical analysis, “a” represents the reference group, “b” represents significantly lower than the “a” group (*p* < 0.05), “c” represents significantly lower than the “b” group (*p* < 0.05), and “d” represents significantly lower than the “c” group (*p* < 0.05).

The expression levels of proinflammatory genes (*COX-2*, *TNF-α*, *IL-1β*, and *IL-6*) were elevated in LPS-stimulated cells (Figure 7). However, treatment with rice seed extracts significantly decreased the expression levels of proinflammatory mRNAs induced by LPS treatment; this was especially observed in cells treated with resveratrol-producing rice seed extracts, namely the germinated rice seed extract (DJ526_5).

### 2.8. Effect of Germinated Rice Seed Extracts on Expression of Inflammatory Proteins

**Figure 8:**
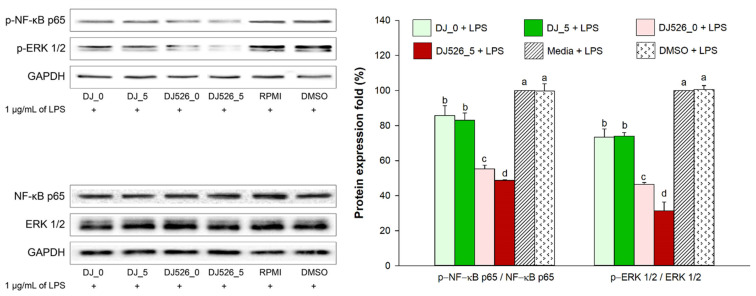
Effect of germinated rice seed extracts on p-NF-κB p65 and p-ERK 1/2 expression in LPS-stimulated adipocytes. The concentrations of treatments and DMSO were 100 µg/mL and 0.1%, respectively. For LPS treatment, “+” represents the treatment of LPS and “−“ represents the nontreatment of LPS. Letters (a–d) indicate significant differences (*p* < 0.05) between DJ_0, DJ_5, DJ526_0, and DJ526_5 (where a > b > c > d). In the statistical analysis, “a” represents the reference group, “b” represents significantly lower than the “a” group (*p* < 0.05), “c” represents significantly lower than the “b” group (*p* < 0.05), and “d” represents significantly lower than the “c” group (*p* < 0.05).

The effect of DJ_0, DJ_5, DJ526_0, and DJ526_5 treatments on the activation of inflammatory pathways was evaluated in LPS-stimulated adipocytes. Figure 8 shows that 1 µg/mL of LPS activated the NF-κB signaling pathway by upregulating p-NF-κB p65 production and the MAPK pathway by increasing the production of p-ERK 1/2. Treatment with resveratrol-producing rice seed extract reduced p-NF-κB p65 and p-ERK 1/2 production compared with normal rice. The five-day-old germinated DJ526 also reduced the production of p-NF-κB p65 and p-ERK 1/2 in LPS-stimulated adipocytes compared with nongeminated DJ526.

## 3. Discussion

Resveratrol- and piceid-enriched rice have been shown to reduce inflammation in RAW264.7 macrophage cells [40]. In the present study, the inhibitory effects of resveratrol-enriched rice seed extract on adipogenic and inflammatory genes and proteins were evaluated in 3T3-L1 preadipocytes and LPS-stimulated adipocytes. The optimal treatment concentration was selected based on cell viability (Figure 1) and lipid accumulation in the cells (Figure 3). Concentrations of transgenic rice seed extract ranging from 10 to 100 µg/mL significantly enhanced the proliferation of 3T3-L1 cells without cytotoxicity. The cell viability ratio slightly decreased upon treatment with higher concentrations of germinated rice seed extract (125 µg/mL). Lipid accumulation was lowest in cells treated with 100 µg/mL of resveratrol-enriched rice seed extract. Therefore, a concentration of 100 µg/mL was selected for further experiments.

Adipogenesis of 3T3-L1 induced by dexamethasone and IBMX [41] activates adipogenic transcription factors, PPARγ and C/EBPα [42]. During the differentiation of 3T3-L1 fibroblasts to adipocytes, PPARγ and C/EBPα regulate the expression of adipogenic genes such as GLUT-4, FAS, and adiponectin [43,44]. Here, the expression of adipogenic transcription factors and genes was significantly higher in MDI-induced cells; however, their expression was remarkably lower in extract-treated cells, especially in those treated with DJ56, which contain higher levels of resveratrol in their seeds. Rayalam, S. et al. [45] also linked resveratrol to the downregulation of C/EBPα, FAS, and SREBP-1c in 3T3-L1 cells. Park, I.S. et al. [46] reported that treatment of resveratrol suppressed the expression of adipogenic markers such as C/EBPα, PPARγ, and adipocyte fatty-acid-binding protein. In addition, lipid accumulation and expression levels of PPARγ, C/EBPα, and SREBP-1c expressions were lower in resveratrol-treated 3T3-L1 adipocytes [47].

The ERK signaling pathway plays a key role in cell differentiation [48]. Sale, E.M. et al. [49] suggested that ERKs orchestrate the differentiation of 3T3-L1 cells into adipocytes. Similarly, Prusty, D. et al. [50] found that the activation of ERK signaling enhances adipogenesis by upregulating the expression of *PPARγ* and *C/EBPα* in MDI-induced 3T3-L1 cells. Oh, J.H. et al. [51] reported that the down-regulation of PPARγ and MAPK pathway proteins (including p-ERK 1/2) led to the inhibition effect on adipogenic differentiation of 3T3-L1 pre-adipocytes. Lee, H. et al. [10] demonstrated that the adipogenesis process can be inhibited by suppressing the phosphorylation of ERK and Akt. The Akt pathway is responsible for adipogenesis [52]. The phosphorylation of Akt induces SREBP-1c activation, leading to the activation of PPARγ and C/EBPs and inducing adipocyte differentiation [53]. Li, N. et al. [4] found that Akt and ERK 1/2 are upregulated in adipogenesis. They also observed that adipogenic markers such as C/EBPs, PPARγ, and adipogenic genes are downregulated when Akt is suppressed, which agrees with our results, as MDI media induced the activation of ERK 1/2 (Figure 5c) and Akt (Figure 5d) during 3T3-L1 cell differentiation. However, DJ526_5 treatment significantly inhibited ERK 1/2 and Akt and, therefore, PPARγ and C/EBPα (Figure 5).

Proinflammatory molecules are expressed to alleviate chronic inflammation during obesity. Kim, H.L. et al. [54] showed that 1 µg/mL of LPS triggers the production of nitric oxide (NO), iNOS mRNA, and NF-κB protein in adipocytes. They also measured increased expression levels of proinflammatory cytokines like IL-1β, IL-6, COX, and TNF-α in LPS-treated adipocytes. In our study, 1 µg/mL of LPS significantly activated the ERK 1/2 and NF-κB signaling pathways and upregulated proinflammatory cytokines in 3T3-L1 adipocytes. Treatment with DJ526_5, which contains a high amount of resveratrol and piceid, may have downregulated the expression of COX-2, TNF-α, IL-1β, and IL-6 by inhibiting the ERK 1/2 and NF-κB signaling pathways. Moreover, the anti-inflammatory activities of resveratrol have been observed in vivo [55]. Ding, S. et al. [55] found that inflammatory factors including CC-chemokine receptor 2, monocyte chemoattractant proteins (MCP)-1, TNF-α, and IL-6, GLUT-4, and insulin receptor substrate 1, significantly decreased in adipose tissue following treatment with resveratrol.

## 4. Materials and Methods

### 4.1. Treatments

**Figure 9:**
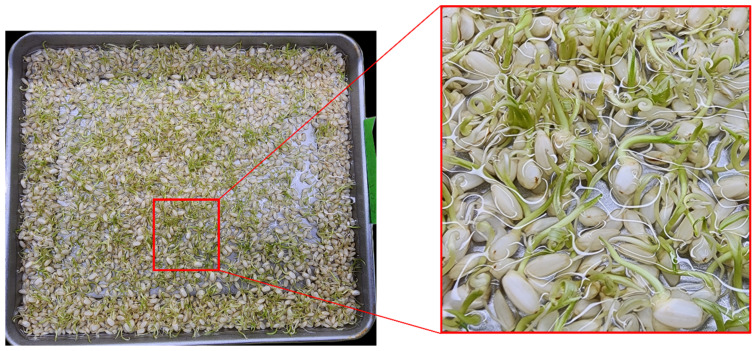
Germinated rice seed. The sterilized unpeeled seeds were allowed to germinate for five days in the sterile distilled water.

The treatments used in this study were the same as those in our previous study [40]. The resveratrol content in DJ_0, DJ_5, DJ526_0, and DJ526_5 is shown in Table S1. The germinated seeds are shown in Figure 9.

### 4.2. 3T3-L1 Cell Culture

Three types of media were used in this experiment: “cell culture media” that includes 10% bovine calf serum (Welgene, Gyeongsangbuk, Republic of Korea) and 1% of penicillin/streptomycin (Hyclone, Logan, UT, USA) in Dulbecco′s modified eagle’s medium (DMEM; Welgene); “MDI media”, which is cell culture media supplemented with 1 μM of dexamethasone (Sigma-Aldrich, Saint Louis, MO, USA), 500 μM of 3-isobutyl-1-methylxanthine (IBMX; Sigma-Aldrich), and 10 μg/mL of insulin (Sigma-Aldrich); and “adipogenic media”, which is cell culture media containing 10 μg/mL of insulin.

**Figure 10:**
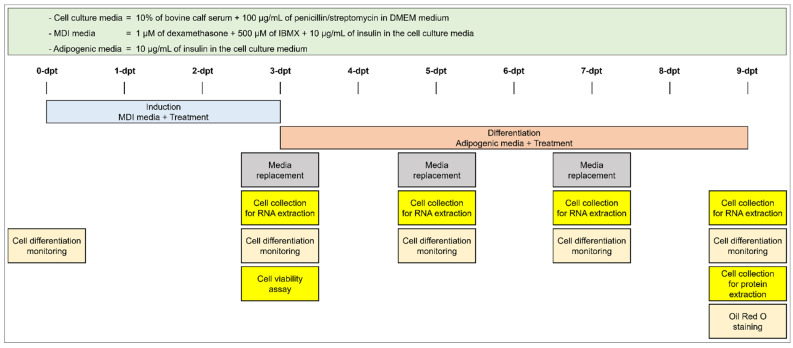
Overview of the experimental procedure and adipogenesis timeline. Dpt: days post treatment.

3T3-L1 cells were cultured in cell culture media at 37 °C and 5% CO_2_. Cells were grown to confluence before an in vitro antiadipogenic or anti-inflammatory assay. The antiadipogenic experiment procedure is described in Figure 10.

### 4.3. 3T3-L1 Cell Viability Assay

Confluent cells were cultured with various concentrations of each treatment in MDI media. After 72 h, the cell viability of the treated cells was determined using EZ-Cytox cell viability solution (DoGenBio, Seoul, Republic of Korea). The reaction was conducted at 37 °C for 4 h. The absorbance at 450 nm (A_450_) was measured using a SpectraMax^®^ ABS Plus Microplate Reader (Molecular Devices, San Jose, CA, USA), and the cell viability ratio was calculated by comparing the A_450_ of the treatment group with that of the media group (untreated).

### 4.4. 3T3-L1 Cell Differentiation

The differentiation of 3T3-L1 cells was induced with MDI media. The first induction day was assigned as 0 days post treatment (dpt). At 3 dpt, the MDI medium was replaced with adipogenic medium and was replaced every two days. During differentiation, cells received 100 μg/mL (final concentration) of each treatment supplemented in the indicated media (MDI or adipogenic media). Differentiation of 3T3-L1 cells was monitored under an IM-3 series microscope (Optika, Ponteranica, Italy) at 0, 3, 5, 7, and 9 dpt.

### 4.5. Oil Red O Staining Assay

At 9 dpt, the cells were fixed with 3.7% formaldehyde (Sigma-Aldrich, Saint Louis, MO, USA) at room temperature for 30 min. The solution was discarded, and the cells were washed with distilled water. Then, 60% isopropanol (100 µL) was added to each well, and the plate was incubated in the dark at room temperature for 5 min. The solution was gently discarded using a pipette. The cells were stained with oil red O solution (Sigma-Aldrich; 0.3% prepared in distilled water) in the dark at room temperature. After 15 min, the cells were washed five times with distilled water until the excess dye was removed completely. The cells were allowed to dry by opening the plate cover at room temperature for 30 min. Stained lipid droplets (red spots) in the cells were imaged under an IM-3 series microscope.

After imaging, 60% isopropanol (200 µL) was added to each well, and the plate was incubated at room temperature for 10 min. The intracellular lipid content was measured using a SpectraMax^®^ ABS Plus at 500 nm. Lipid accumulation was calculated according to the following formula:

(1) $$\mathrm{Lipid}\;\mathrm{accumulation}\;(\%)=\frac{\mathrm{Absorbance}\;\mathrm{at}\;500\;\mathrm{nm}\;\mathrm{of}\;\mathrm{test}\;\mathrm{group}\;-\;\mathrm{Absorbance}\;\mathrm{at}\;500\;\mathrm{nm}\;\mathrm{of}\;\mathrm{blank}}{\mathrm{Absorbance}\;\mathrm{at}\;500\;\mathrm{nm}\;\mathrm{of}\;\mathrm{control}\;\mathrm{group}\;-\;\mathrm{Absorbance}\;\mathrm{at}\;500\;\mathrm{nm}\;\mathrm{of}\;\mathrm{blank}}\;\times \;100,$$

### 4.6. RNA Extraction and cDNA Synthesis

Total RNA was extracted using TRI Reagent™ solution (Invitrogen, Waltham, MA, USA) and quantified using a SpectraDrop™ microvolume microplate (Molecular Devices). The ratio of the absorbance at 260 nm and 280 nm (A260:A280) and the absorbance at 260 nm and 230 nm (A260:A230) of the extracted RNA was in the acceptable range (1.8–2.0). The first strand of cDNA was synthesized from 1 µg of total RNA using a power cDNA synthesis kit (iNtRON Biotechnology, Inc., Gyeonggi, Republic of Korea).

### 4.7. Real-Time PCR Analysis

The mRNA expression levels of adipogenic genes were evaluated using a Bio-Rad CFX Maestro system. The qPCR reaction consisted of a 5 ng cDNA template and 0.375 µM of specific primers (Table 1) in RealMOD™ Green W^2^ 2× qPCR mix (iNtRON Biotechnology, Inc., Burlington, MA, USA). The PCR reaction included 5 ng of cDNA template and 0.375 µM of each specific primer and was conducted as follows: predenaturation (95 °C for 10 min), 40 cycles of PCR (denaturation: 95 °C for 20 s; annealing: 60 °C for 20 s; extension: 72 °C for 30 s); and final extension (72 °C for 5 min). Gene expression levels were calculated using the 2^−ΔΔCt^ [56] method, and *β-actin* was used as the reference gene.

### 4.8. Western Blot Analysis

The Western blot assay was carried out as described by Monmai, C. et al. [57]. The concentrations of proteins diluted 1:40 in lysis buffer were measured using Bradford reagent (Welgene) and determined from a standard curve of bovine serum albumin (BSA; Sigma-Aldrich) (Figure S1). The following primary antibodies were incubated at 4 °C overnight: PPARγ (1:2000; Cell Signaling, Danvers, MA, USA), C/EBPα (1:2000; Cell Signaling), p-ERK 1/2 (1:2000; Cell Signaling), ERK 1/2 (1:2000; Cell Signaling), p-Akt (1:2000; Cell Signaling), Akt (1:2000; Santa Cruz Biotechnology, Dallas, TX, USA), p-NF-κB p65 (1:2000; Cell Signaling), NF-κB p65 (1:2000; Santa Cruz Biotechnology), and GAPDH (1:5000; Santa Cruz Biotechnology). Secondary antibodies were incubated at room temperature for 2 h [goat anti-rabbit IgG(H + L)-HRP (1:5000; GenDepot, Baker, TX, USA) or m-IgGκ BP-HRP (1:5000; Santa Cruz Biotechnology)]. Protein signaling was detected using Clarity Max™ Western ECL Substrate (Bio-Rad, Hercules, CA, USA). The detected signaling was imaged and quantified using a ChemiDoc Imaging System (Bio-Rad).

### 4.9. Anti-Inflammatory Adipocyte Assays

The anti-inflammatory activities of resveratrol-producing rice seed extracts were evaluated in differentiated-3T3-L1 adipocytes cells as described by Yamamoto, K. et al. [58] and Kim, H.L. et al. [54] with some modifications (Figure S2). Briefly, the differentiated-3T3-L1 adipocytes were pretreated with various concentrations of each treatment for 1 h. The cells were then stimulated with 1 µg/mL of LPS. After 6 h, total RNA was extracted, and cDNA was synthesized as described in Section 4.6. Inflammatory gene expression was quantified as described in Section 4.7. Primer sequences are shown in Table 2. After 24 h of stimulation, the viability of cells was measured as described in Section 4.3, and Western blots were analyzed as described in Section 4.8. The calibration curve for protein quantification is shown in Figure S3.

### 4.10. Statistical Analysis

Experimental data are presented as mean ± standard deviation. Statistix8.1 software (Statistix, Tallahassee, FL, USA) was used for statistical analysis. Significant differences were determined using one-way analysis of variance followed by Duncan’s multiple-range tests. Differences were considered significant if *p* < 0.05.

## 5. Conclusions

In summary, the antiadipogenic and anti-inflammatory effects of resveratrol-enriched rice seed were evaluated in 3T3-L1 adipocytes. The activation of adipogenic transcription factors (PPARγ and C/EBPα) and adipogenic signaling pathways (ERK 1/2 and Akt) was inhibited in cells treated with resveratrol-enriched rice seed extract. This decreased lipid accumulation and expression of adipogenic genes in these cells. Furthermore, LPS-induced proinflammatory responses were significantly lower in resveratrol-enriched rice seed extract-treated adipocytes due to suppression of the ERK and NF-κB signaling pathways. Future studies should explore how resveratrol-enriched rice seeds affect adipogenesis and inflammation in vivo. Ultimately, resveratrol-enriched rice could be used to attenuate adipogenesis and obesity-induced inflammation.

## Figures and Tables

**Table 1:** Sequences of the primers in the antiadipogenic assay.

Target Gene	Nucleotide Sequences	Accession No.
Adiponectin	FP: 5′–AAA GGA GAG CCT GGA GAA GC–3′ RP: 5′–GTA GAG TCC CGG AAT GTT GC–3′	NM_009605.5
*C/EBPα*	FP: 5′–TTA CAA CAG GCC AGG TTT CC–3′ RP: 5′–AAC TCC AGT CCC TCT GGG AT–3′	NM_001287514.1
*FAS*	FP: 5′–CTC TGA TCA GTG GCC TCC TC–3′ RP: 5′–TGC TGC AGT TTG GTC TGA AC–3′	AF127033.1
*PPARγ*	FP: 5′–CCC TGG CAA AGC ATT TGT AT–3′ RP: 5′–GAA ACT GGC ACC CTT GAA AA–3′	AB644275.1
*SREBP-1*	FP: 5′–AGC TCA AAG ACC TGG TGG TG–3′ RP: 5′–TCA TGC CCT CCA TAG ACA CA–3′	BC056922.1
*β-actin*	FP: 5′–CCA CAG CTG AGA GGG AAA TC–3′ RP: 5′–AAG GAA GGC TGG AAA AGA GC–3′	NM_007393.5

FP represents the forward primer, and RP represents the reverse primer.

**Table 2:** Sequences of the primers used in the anti-inflammatory assay.

Target Gene	Sequence (5′–3′)	Accession No.
*COX-2*	FP: 5′–AGA AGG AAA TGG CTG CAG AA–3′ RP: 5′–GCT CGG CTT CCA GTA TTGAG–3′	NM_011198.4
*TNF-α*	FP: 5′–ATG AGC ACA GAA AGC ATG ATC–3′ RP: 5′–TAC AGGCTT GTC ACT CGA ATT–3′	D84199.2
*IL-1β*	FP: 5′–GGG CCT CAA AGG AAA GAA TC–3′ RP: 5′–TAC CAG TTG GGGAAC TCT GC–3′	NM_008361.4
*IL-6*	FP: 5′–AGT TGC CTT CTT GGG ACT GA–3′ RP: 5′–CAG AAT TGC CAT TGC ACA AC–3′	NM_031168.2
*β-actin*	FP: 5′–CCA CAG CTG AGA GGG AAA TC–3′ RP: 5′–AAG GAA GGC TGG AAA AGA GC–3′	NM_007393.5

FP represents the forward primer and RP represents the reverse primer.

## Data Availability

All the applicable data have been provided in the manuscript.

## Supplementary Materials

The following supporting information can be downloaded at: https://www.mdpi.com/article/10.3390/molecules28155750/s1, Table S1: The piceid and resveratrol content in each extract; Figure S1: BSA calibration curve for protein quantification (antiadipogenic assay); Figure S2: Overview of the anti-inflammation experimental procedure in LPS-stimulated adipocytes; Figure S3: BSA calibration curve for protein quantification (anti-inflammatory assay).

## Sample Availability

Samples of the compounds DJ_0, DJ_5, DJ526_0, and DJ526_5 are available from the authors.
