# Supplementary material for: Germinated Rice Seeds Improved Resveratrol Production to Suppress Adipogenic and Inflammatory Molecules in 3T3-L1 Adipocytes
Source: Molecules. 2023 Jul 29;28(15):5750. doi: 10.3390/molecules28155750 (PMC10420918; doi:10.3390/molecules28155750)
Supplement: Supplementary file 1 [file molecules-28-05750-s001.zip › molecules-2514719-supplementary.pdf]

Supplementary information

Table S1. The piceid and resveratrol content in each extract.

Extracts	Piceid (µg/g dry weight)	Resveratrol (µg/g dry weight)
DJ_0	Not detectable	Not detectable
DJ_5	Not detectable	Not detectable
DJ526_0	4.724 ± 0.023	2.605 ± 0.001
DJ526_5	16.879 ± 0.024	3.230 ± 0.600

Figure S1. BSA calibration curve for protein quantification (antiadipogenic assay)

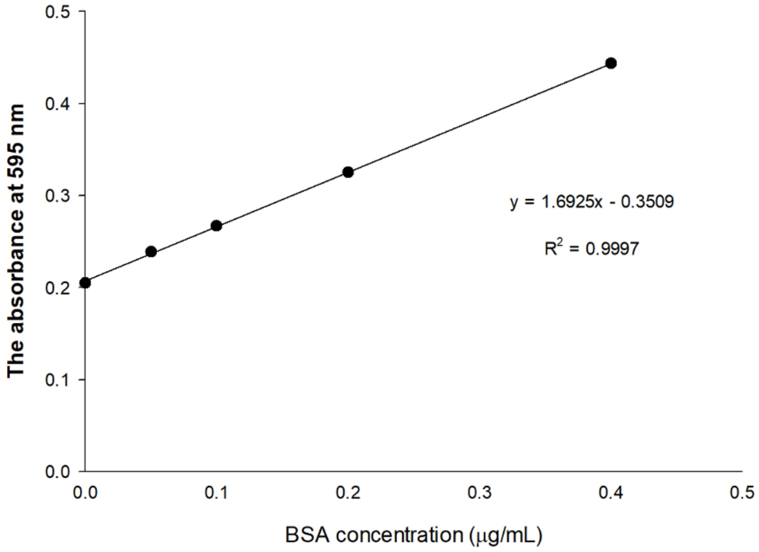

BSA concentration (µg/mL)	Mean absorbance
0.40	0.443
0.20	0.325
0.10	0.267
0.05	0.239
0.00	0.205

Figure S2. Overview of the anti-inflammation experimental procedure in LPS-stimulated adipocytes.

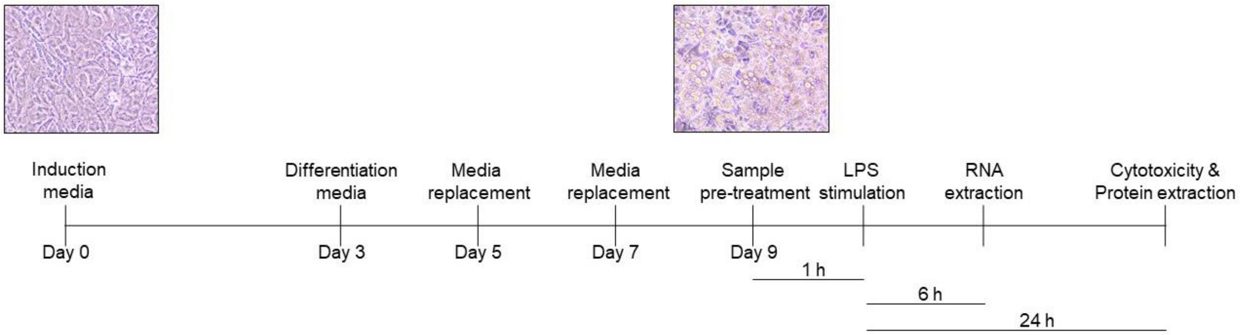

Figure S3. BSA calibration curve for protein quantification (anti-inflammatory assay)

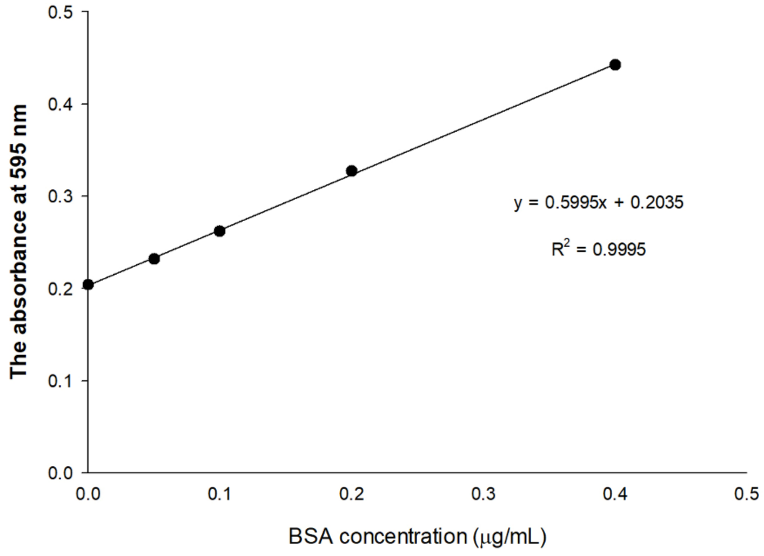

BSA concentration (µg/mL)	Mean absorbance
0.40	0.442
0.20	0.327
0.10	0.262
0.05	0.232
0.00	0.204
